# Analysis of prevalence and associated biomarkers of sarcopenia in older adults living in rural community in Wuhan, China

**DOI:** 10.3389/fendo.2025.1635234

**Published:** 2025-09-03

**Authors:** Yun Zhou, Kemeng Zhang, Yanling Zhang, Ping He, Zhaohui Wang

**Affiliations:** Department of Geriatrics, Union Hospital, Tongji Medical College, Huazhong University of Science and Technology, Wuhan, China

**Keywords:** sarcopenia, disease incidence, biomarkers, older adults, rural community

## Abstract

**Background:**

Sarcopenia is a disease of loss of muscle mass and decreased skeletal muscle function with impaired ability in the activities of daily life. Exploring potential biomarkers for sarcopenia may lead to early screening and intervention for sarcopenia.This study investigated the prevalence and potential biomarkers of sarcopenia in older adults living in rural community in Wuhan,China.

**Methods:**

This cross-sectional study involved 236 older participants (age ≥65 years) who received a health examination that included body composition and 23 circulating biomarkers. Sarcopenia was defined by the Asian Working Group for Sarcopenia revised in 2019 (AWGS2019). We divided the participants into a non-sarcopenia group and a sarcopenia group. The correlation between biomarkers and sarcopenia was analyzed by independent sample *t*-test, and then the significant variables of the *t*-test (*p* < 0.05) were included in the binary logistic regression model to determine the independent factors associated with sarcopenia.

**Results:**

Among the 236 participants, 61% were females, with a mean age of 70.6 ± 4.4years. The prevalence of sarcopenia in rural community was 25.4%(men 20.7%, women 28.5%). Analyses were conducted using binary logistic regression, growth differentiation factor 11(GDF11), was an independent risk factor for sarcopenia [Exp (B) 1.031, 95% CI: 1.010-1.052, *p* = 0.003]. Body mass index(BMI), albumin(ALB), fibroblast growth factor 19(FGF19), and tumour necrosis factor alpha(TNF-α) were independent protective factors for sarcopenia [BMI: Exp (B) 0.007, 95% CI: 0.000-0.244, *p* = 0.006;ALB: Exp (B) 0.490, 95% CI: 0.281-0.853, *p* = 0.012; FGF19: Exp(B) 0.804, 95% CI: 0.683-0.946, *p* = 0.009; TNF-α: Exp (B) 0.379, 95% CI: 0.194-0.742, *p* = 0.005].

**Conclusions:**

About a quarter of older adults in rural Chinese communities are at risk of sarcopenia. Lower BMI, lower serum ALB, FGF19, TNF-α, and higher circulating GDF11 are associated with sarcopenia.

## Background

Sarcopenia is a geriatric disease characterized by age-related decline in skeletal muscle mass and muscle strength or physical function (1). According to the national statistics of China in 2021, the older adults population aged 65 and above has increased to 190 million, accounting for about 13.5% of the total population (2). China is facing severe challenges related to population aging. Chronic diseases such as age-related sarcopenia have a huge impact on family medical burden and social public health expenditure. At present, there are limited epidemiological information regarding sarcopenia, especially those on the older adults living in the rural community of China (3, 4).

The etiology and pathogenesis of sarcopenia are complex and varied, and there may be multiple interactions, such as neuromuscular degeneration, chronic inflammation, oxidative stress response and changes in the secretion of anabolic hormones (5, 6). Currently, standardized diagnostic criteria for sarcopenia have not been determined internationally, and different populations, measuring devices and means have a great impact on the diagnosis of sarcopenia. However, exploring biomarkers related to sarcopenia can quickly and economically support the diagnosis of sarcopenia. Therefore, people’s interest in exploring biomarkers related to sarcopenia is gradually increasing. Although some biomarkers, such as albumin (ALB), interleukin 6 (IL-6), growth differentiation factor 11 (GDF11), and recently the emerging fibroblast growth factor 19 (FGF19), have been considered as biomarkers related to sarcopenia, the types and mechanisms of these biomarkers have not been clearly defined and accepted internationally (7, 8). Additionally, some studies suggest that FGF19, as a potential target for treating metabolic diseases, may be a promising new factor in the treatment of sarcopenia (9). According to reports, GDF11, as a member of the transforming growth factor-beta (TGF-β) superfamily, can alleviate functional impairments in age-related diseases (10). Some studies have shown that chronic inflammation associated with aging may cause imbalance of protein synthesis and metabolism, leading to muscle loss (11–13), but there are still other studies with contradictory results (14). We referred to UK Biobank and other relevant literature, and considered that the occurrence of sarcopenia was related to mechanisms such as inflammation and oxidative stress, so a total of 23 biomarkers were selected. They were biomarkers such as inflammatory factors TNF-α, IL-6, sex hormones such as dehydroepiandrosterone (DHEA), and FGF19, GDF11, growth differentiation factor 15 (GDF15), insulin-like growth factor 1 (IGF-1), as well as some laboratory indicators that are relatively easy to obtain clinically such as liver and kidney function related biomarkers alanine aminotransferase (ALT), aspartate aminotransferase (AST), direct bilirubin (DBIL), total bilirubin (TBIL), ALB, alkaline-phosphatase (ALP), gamma-glutamyl transferase (γ-GT), uric acid (UA), creatinine (CREA) and blood lipid and blood glucose related biomarkers glucose (GLU), triglycerides (TG), total cholesterol (TC), high-density lipoprotein (HDL), low-density lipoprotein (LDL), glycosylated serum protein (GSP), adiponectin (ADP). Based on the data we were able to collect, we ultimately chose these 23 biomarkers for analysis (15). The relationship between these biomarkers related to sarcopenia were analyzed using data from rural communities in China.

The main objective of this study was to verify the potential biomarkers related to sarcopenia in the older adults in rural communities of China, the secondary goal was to estimate the incidence rate of sarcopenia in the older adults in rural China.

## Methods

### Participants and study design

This study is a single-center rural community-based cross-sectional study. The older adults of our study were residents of Huangling Community and Fenghuang Yuan Community in Wuhan, China. From September 26, 2021 to October 30, 2021, the participants had taken the routine health examination in Junshan Health Center, Wuhan. The inclusion criteria are as follows: 1. sign informed consent form and promise to comply with research procedures and cooperate in implementing the entire research process; 2. age ≥ 65 years old; 3. stable health condition. Participants were excluded from the study if they were: 1. unable to communicate with the investigator or refused to sign the informed consent; 2. the participants were unable to complete a series of tests such as height, weight, body composition, handgrip strength and 6-meter gait speed test; 3. patients with malignant tumors; 4. myocardial infarction or severe heart failure; 5. other serious comorbidities, such as renal failure during hemodialysis, severe mental illnesses, such as bipolar disorder, schizophrenia; 6. difficult in mobility, e.g, unable to walk without wheelchairs or other assistive devices, or patients with prosthetics or amputations. The total number of people who underwent health examinations in 2021 was 571. Based on the exclusion criteria established, the final 236 participants (92 men and 144 women) met the inclusion criteria. The protocol of this study was approved by the ethics committee of Union Hospital, Tongji Medical College, Huazhong University of Science and Technology (UHCT-IEC-SOP-016-03-01) and followed the tenets of the Helsinki Declaration.

### Sample size calculation

For the sample size calculation of this cross-sectional study, the estimated prevalence of sarcopenia was 13.5%, α=0.05, precision 10%. A two-sided confidence interval was used and the sample size was calculated as N = 198 by PASS 11 software. Considering the 10% loss rate, at least 220 patients should be included in this study. According to our set into the exclusion criteria, this study finally included in the number of cases to 236.

### Body composition

We used the 8-contact multi-frequency electrode bioelectrical impedance analysis (BIA) device (BCA-2A, TFHT, Beijing, China) to measure the body composition. It has five different measurement frequencies (5, 50, 100, 250 and 500 kHz). Skeletal muscle content (ASM) of extremities was the sum of skeletal muscle content of both upper and lower limbs (16). Skeletal muscle mass normalized for height (RASM, ASM/Ht^2^) was defined as the ratio of ASM (kg) to height square (m^2^) (17). The ASM/Ht^2^ < 7.0kg/m^2^ in men or < 5.7kg/m^2^ in women was diagnosed as skeletal muscle loss.

### Handgrip strength

Handgrip strength was measured using the standard Adjustable Digital Grip Strength Tester (CSTF-WL, TFHT, Beijing, China). Participants were asked to perform two maximum handgrip strength tests with their dominant hand for data analysis with the best results (18, 19). The criteria for low handgrip strength was less than 28kg for men or less than 18kg for women.

### Gait speed

A digital gait speed tester (CSTF-6MBS, TFHT, Beijing, China) was used to measure gait speed (m/s). Participants were asked to perform the test at their usual gait speed on a 6-meter track. If participants preferred using a walker, they were allowed to use a walker during the test. Slow gait speed was defined as a gait speed < 1.0m/s (20).

### Anthropometry

Height and weight were measured using a digital height and weight tester (CSTF-ST, TFHT, Beijing, China). Participants were asked to remove their shoes during measurement. Body mass index (BMI) was calculated by dividing their weight by the square of their height (kg/m^2^).

### Definition of sarcopenia

We identified patients with sarcopenia using the AWGS2019 sarcopenia consensus. The diagnosis of sarcopenia required ASM/Ht^2^ < 7.0 kg/m^2^ in male and < 5.7 kg/m^2^ in female; handgrip strength < 28kg in male and < 18kg in female and/or gait speed < 1.0m/s (20).

### Laboratory testing

Fasting blood samples were collected by venipuncture and centrifuged within 1 hour after sampling. Fifteen parameters including ALT, AST, DBIL, TBIL, ALB, ALP, γ-GT, CREA, UA, GLU, TG, TC, HDL, LDL and GSP were analyzed. Eight biomarkers including ADP, FGF19, GDF11, GDF15, IGF-1, TNF-α, IL-6 and DHEA were determined by human enzyme-related immunosorbent assay kit (ELISA) (ELK Biotechnology CO., LTD).

### Statistical analysis

SPSS 25.0 software was used to analyze the data. Continuous variables and categorical variables were expressed as mean ± SD and percentage, respectively. For the character profiles we used ANOVA, for *post-hoc* analyses, LSD test was used when Homogeneity of variance and Tamhane’s T2 test was used when heterogeneity of variance. Independent sample t-test was used for circulating biomarker tests. Mann-Whitney U-test was used when the variables did not followed a normal distribution. The independent association factors of sarcopenia were analyzed by multiple logistics regression analysis, and the statistical test was two-tail test. *p*<0.05 was considered to be statistically significant.

## Result

### Clinical characteristics

The participant characteristics are shown in **Table 1**. During the data collection period from September 26, 2021 to October 30, 2021, a total of 236 older adults (aged ≥65 years) from rural communities in Wuhan participated in the sarcopenia screening program, including 92 males and 144 females. A total of 60 older adults were diagnosed with sarcopenia according to the 2019AWGS diagnostic criteria, including 19 males and 41 females. The prevalence of sarcopenia was 25.4%. The prevalence was 20.7% in older adult males and 28.5% in older adult females.

**Table 1:** Demographic characteristics and physical examination measures of 236 older adults living in rural community in Wuhan, China.

Variable	Total (*n*=236)	Non-sarcopenia (*n*=176)		Sarcopenia (*n*=60)		F-value	*P*-value
		Male(n=73)	female(n=103)	Male (n=19)	female(n=41)	F	*P*
Age (years)	70.6±4.4	71.0±4.4	70.2±4.2	72.2±5.1	70.0±4.7	1.614	0.187
BMI (kg/m^2^)	23.8±3.4	25.2±3.0	25.1 ±2.5	19.6±1.8	20.3 ±1.8	59.035	<0.001^ab^
GS (m/s)	0.86±0.19	0.87±0.21	0.87 ±0.20	0.79±0.13	0.84 ±0.18	1.254	0.291
HS (kg)	23.0±8.3	31.3±7.6	18.8 ±5.1	24.7±6.4	17.8 ±4.5	74.713	<0.001^acd^
ASM/Ht^2^ (kg/m^2^)	6.81±1.38	8.23±1.04	6.61 ±0.80	6.27±0.51	5.01 ±0.58	137.882	<0.001^abcd^
Upper limb fat (kg)	2.54±0.98	2.49±0.67	3.00 ±0.82	1.36±0.29	2.06 ±1.28	26.580	<0.001^abcd^
Lower limb fat (kg)	6.09±1.85	6.15±1.59	7.12 ±1.49	3.34±0.75	4.65 ±1.12	55.865	<0.001^abcd^
Upper limb muscles (kg)	4.22±1.08	5.38±0.82	4.02 ±0.55	3.77±0.43	2.86 ±0.49	152.445	<0.001^abcd^
Lower limb muscles (kg)	12.81±3.37	16.80±2.38	11.62 ±1.44	12.50±1.37	8.85 ±1.34	208.212	<0.001^abcd^
PBF (%)	28.8±6.3	25.2±4.3	33.6 ±4.2	18.7±3.1	27.5 ±4.5	98.018	<0.001^abcd^

^a^Differences between the male without sarcopenia and male with sarcopenia groups, ^b^Differences between the female without sarcopenia and female with sarcopenia groups, ^c^Differences between the male without sarcopenia and female without sarcopenia groups, ^d^Differences between the male with sarcopenia and female with sarcopenia groups.

BMI, body mass index; HS, handgrip strength; GS, gait speed; ASM/Ht^2^, appendicular skeletal muscle mass, appendicular soft lean mass/(height)^2^; PBF, percentage body fat.

The diagnosis of sarcopenia requires ASM/Ht^2^ < 7.0 kg/m^2^ in male and < 5.7 kg/m^2^ in female, grip strength < 28kg in male and < 18kg in female and/or gait speed < 1.0m/s.

### Variables associated with sarcopenia in rural community older adults

**Table 1** presents the clinical characteristics of the old adults by sex and sarcopenia and non-sarcopenia groups. BMI, handgrip strength, ASM/Ht^2^, fat content and muscle content of the upper and lower limbs and body fat percentage of old adults in sarcopenia group were significantly lower than those in non-sarcopenia group (*p* < 0.001). Compared with male non-sarcopenia groups, male sarcopenia groups had lower BMI, ASM/Ht^2^, fat content and muscle content of the upper and lower limbs and body fat percentage, the same was for comparisons between female non-sarcopenia group and female sarcopenia group. Handgrip strength was lower in the male sarcopenia group than in the male non-sarcopenia group, but not in the female group. Both in the sarcopenia and non-sarcopenia groups, the female group had lower handgrip strength, ASM/Ht^2^ and muscle mass of upper and lower limbs, and higher upper and lower limb fat mass than the male group.

The circulating biomarker tests of the participants are shown in **Table 2**. *t*-test or U-test analysis revealed differences in circulation markers older adults with and without sarcopenia. Compared with circulating factors in non-sarcopenia older adults, FGF19 and GDF11 were significantly higher in sarcopenia older adults. The circulating factors in sarcopenia group were significantly lower: ALT, AST, ALB, γ-GT, CREA, UA, GLU, TG, LDL, GDF15, TNF-α and IL6. There were no significant differences in other circulating factors (DBIL, TBIL, ALP, TC, HDL, GSP, ADP, IGF-1 and DHEA) between non-sarcopenia and sarcopenia groups (*p* ≥ 0.05).

**Table 2:** Characteristics of biomarkers in 236 older adults living in rural community in Wuhan, China.

Variable	Non-sarcopenia	Sarcopenia	*t*/z	*P*-value
ALT (U/L)	18.84±11.72	12.57±6.69	-4.795	<0.001*
AST (U/L)	25.08±9.56	22.42±6.94	-2.360	0.018*
DBIL (μmol/L)	9.82±2.72	9.75±2.67	0.185	0.853
TBIL (μmol/L)	11.80±3.50	11.83±3.41	-0.021	0.983
ALB (g/L)	36.60±3.68	35.45±4.00	2.047	0.042*
ALP (U/L)	80.82±26.66	79.43±28.67	0.342	0.732
γ-GT (U/L)	33.15±35.95	25.66±35.12	-3.992	<0.001*
CREA(μmol/L)	64.81±25.57	57.94±14.38	-2.115	0.034*
UA (μmol/L)	278.49±79.24	239.54±77.71	3.304	0.001*
GLU (mmol/L)	4.58±1.02	4.29±0.92	1.990	0.048*
TG (mmol)	0.99±0.52	0.76±0.40	3.111	0.002*
TC (mmol)	4.14±0.79	4.06±0.77	0.719	0.473
HDL (mmol)	1.04±0.29	1.10±0.35	-1.325	0.186
LDL (mmol)	2.65±0.71	2.44±0.72	2.005	0.046*
GSP (mmol/L)	1.81±0.30	1.85±0.30	-0.884	0.377
ADP (μg/mL)	9.46±2.70	9.34±1.76	0.402	0.688
FGF19 (pg/mL)	111.08±25.09	124.49±18.75	-4.367	<0.001*
GDF11 (ng/mL)	191.97±142.53	417.33±214.55	-7.375	<0.001*
GDF15 (ng/mL)	0.75±0.48	0.54±0.35	-3.232	0.001*
IGF-1 (ng/mL)	383.80±96.33	376.55±62.43	0.668	0.505
TNFa (pg/mL)	25.00±13.06	16.18±7.22	-4.671	<0.001*
IL6 (pg/mL)	5.59±3.20	3.19±2.64	-5.575	<0.001*
DHEA (ng/mL)	574.74±193.38	550.28±246.49	0.699	0.487

ALT, alanine aminotransferase; AST, aspartate aminotransferase; DBIL, direct bilirubin; TBIL, total bilirubin; ALB, albumin; ALP, alkaline-phosphatase; γ-GT, gamma-glutamyl transferase; CREA, creatinine; UA, uric acid; GLU, glucose; TG, triglycerides; TC, total cholesterol; HDL, high-density lipoprotein; LDL, low-density lipoprotein; GSP, glycosylated serum protein; ADP, adiponectin; FGF19, fibroblast growth factor 19; GDF11, Growth differentiation factor 11; GDF15, growth differentiation factor 15; IGF-1, insulin-like growth factor 1; TNF-α, tumour necrosis factor alpha; IL-6, interleukin 6; DHEA, dehydroepiandrosterone. *, P-value <0.05.

### Independent factors associated with sarcopenia in rural community older adults

In order to further elaborate the related factors of sarcopenia, we included the 15 significantly variables: BMI, FGF19, GDF11, ALT, AST, ALB, γ-GT, CREA, UA, GLU, TG, LDL, GDF15, TNF-α and IL6 from the above univariate analysis into the binary analysis, an analysis of multiple determinants of the sarcopenia was conducted using a stepwise conditional logistic regression with *p* < 0.05 (**Table 3**). The results showed that GDF11 was an risk factor for sarcopenia. BMI, ALB, FGF19 and TNF-α were independent protective factors (**Table 3**). In addition, LDL was not an independent factor in the development of sarcopenia (*p* > 0.05).

**Table 3:** Binary regression analysis of biomarkers associated with sarcopenia in older adults living in rural community in Wuhan, China.

	B	SE	Wald	df	*p*	Exp(B)	95%CI
BMI	-5.015	1.839	7.437	1	0.006	0.007	0.000-0.244
ALB	-0.714	0.283	6.350	1	0.012	0.490	0.281-0.853
LDL	1.709	0.889	3.699	1	0.054	5.522	0.968-31.508
FGF19	-0.218	0.083	6.875	1	0.009	0.804	0.683-0.946
GDF11	0.030	0.010	8.773	1	0.003	1.031	1.010-1.052
TNF-α	-0.970	0.343	8.024	1	0.005	0.379	0.194-0.742

BMI, body mass index; ALB, albumin; LDL, low-density lipoprotein; FGF19, fibroblast growth factor 19; GDF11, Growth differentiation factor 11; TNF-α, tumour necrosis factor alpha. B, regression coefficient; SE, standard error; Wald, Wald statistic; df, degrees of freedom; p, p-value; Exp(B), odds ratio; 95%CI, 95% confidence interval.

## Discussion

In this study, the prevalence of sarcopenia was 25.4% (20.7% in men and 28.5% in women) in the rural older adults in China. In recent years, epidemiological survey results of sarcopenia in Chinese population showed that the prevalence rate of sarcopenia in older adults people aged 60 and above is 3.1%-62.9% (21). A study in Taiwan showed that the prevalence of sarcopenia was 18.6% in 302 aged 65 and older men and 23.0% in women, which is similar to our results (22). A New Mexico senior health survey found that the prevalence of sarcopenia in older men and women was 28.5% and 33.9%, respectively (23). However, other studies have reported low prevalence of sarcopenia, and differences in prevalence results may be due to differences in the definition of sarcopenia, diagnostic cut-off values, or ethnic background of the population.

The older adults with sarcopenia had a lower BMI than the non-sarcopenia older adults. In our study, a total of 60 old adults with sarcopenia were included, and none of which had a BMI greater than 27.9 kg/m^2^, which allowed us to exclude obesity as a confounding factor in our analysis. Our finding that a lower BMI was a risk factor for sarcopenia was consistent with the findings of multiple previous studies suggesting that the higher the BMI in older adults, the lower the risk of sarcopenia (7, 24). While obesity is considered a risk factor for many adverse outcomes, in older adults, being slightly overweight may be beneficial. Studies have also shown that older men were resistant to the dangers of overweight and obesity. Mild overweight, obesity, and even central obesity were beneficial for survival (8). Similarly, a study of hospitalized older adults found that fat mass was associated with a lower risk of death or complications (11). In addition, studies have shown that BMI is positively correlated with muscle mass and fat mass (12–14). It has been pointed out that fat is an energy store for the older adults and helps individuals survive in disease or poor physical condition. The amount of fat can affect lean body mass, and people with high fat amount may have a higher protein intake, which is a protective mechanism against muscle loss (25, 26). Given this, high BMI might play a protective role against loss of muscle mass and strength in older adults. The results of this study further support the negative association between BMI and sarcopenia, so maintaining a high BMI in older adults may help maintain muscle mass and strength.

Studies have shown that the serum albumin of the older adults with sarcopenia is lower than that of the non-sarcopenia older adults participants (27, 28). A meta-study of sarcopenia involving 4,904 community-living and institutional older adults (68-87.6 years) from 9 countries and another meta-analysis of 4,071 participants from 5 countries showed that regardless of age and setting, albumin is negatively associated with sarcopenia, which is easier and less costly to implement than other approaches and ensures timely assessment and early intervention in older adults patients with sarcopenia, Therefore, serum albumin decline can be considered as a reference indicator for biomarkers of sarcopenia. However, albumin concentrations are also affected by processes such as inflammation and infection, so there is no consensus on optimal limits and reference ranges for serum values to assess nutritional status in older adults (29–31). Albumin is the most abundant plasma protein in the body, and its basic role is to regulate the passage of water and solutes through capillaries by maintaining colloidal pressure in the vascular system. ALB dysfunction contributes to sarcopenia pathogenesis through impaired nutrient signaling and amplified oxidative stress. Hypoalbuminemia reduces substrate availability for mammalian target of rapamycin complex 1 (mTORC1)-mediated protein synthesis, while diminished free radical scavenging capacity activates nuclear factor kappa B (NF-κB)-dependent proteolysis, collectively accelerating muscle atrophy (32, 33). Albumin has long been considered integral to the assessment of nutritional status, it is considered to be a very important plasma protein in the assessment of nutritional status, reducing it can alter wound healing, cause immune problems, and reduce lean body mass (34). Improving the nutritional status of the older adults can increase the serum albumin content, which may play a certain role in the treatment of sarcopenia in the older adults.

In older adults with sarcopenia, serum GDF11 levels were significantly elevated compared to non-sarcopenic controls, while GDF15 showed no statistical difference. GDF11 and GDF15 are members of the transforming growth factor-β superfamily, with GDF11 structurally closely related to myostatin (MSTN) (35). Notably, these growth differentiation factors demonstrate opposing pathophysiological effects: GDF11 overexpression paradoxically induces muscle wasting through activin receptor type IIB (ActRIIB)-SMAD2/3 hyperactivation (36), whereas GDF15 consistently promotes sarcopenia via glial cell line-derived neurotrophic factor receptor alpha-like (GFRAL)-mediated appetite suppression and energy imbalance. This mechanistic divergence aligns with receptor specificity—while GDF15 utilizes GFRAL (a Ret tyrosine kinase co-receptor) explaining its classification in GDNF-like families (37, 38), GDF11 signals primarily through ActRIIB.

Multiple studies confirm GDF11 inhibits skeletal muscle regeneration (10, 36, 39). Juli E. Jones recently demonstrated that elevated GDF11 induces cachexia in mice, reducing muscle mass, food intake and body weight while upregulating plasma GDF15. Critically, blocking GFRAL (GDF15 receptor) alleviated anorexia without preventing muscle loss, whereas ActRIIB (GDF11 receptor) inhibition prevented atrophy and improved anorexia (39). Clinically, serum GDF15 >1,200 pg/mL predicts 3.4-fold higher sarcopenia risk independent of comorbidities (37, 40). Therefore, elevated GDF11 constitutes an independent predictor of sarcopenia, operating through direct catabolic signaling distinct from GDF15’s metabolic effects.

We also found that FGF19 was lower in the sarcopenia group than in the non-sarcopenia group. FGF19 is a member of the FGF family, a family of proteins involved in differentiation, development and metabolism (41). FGF19 is similar to endocrine hormone and is secreted by ileum intestinal epithelial cells. The pathogenesis of sarcopenia is related to skeletal muscle metabolism, and FGF19 has been found to play a role in muscle metabolism (42). FGF19 exhibits dual regulation of muscle homeostasis. Although acute FGF19-FGFR4/β-Klotho signaling enhances adenosine monophosphate-activated protein kinase (AMPK)-driven glucose utilization, chronic elevation induces insulin-like growth factor 1 (IGF-1) resistance that disrupts myocyte hypertrophy—a key process in sarcopenia progression (43). Therefore, FGF19 may be an emerging factor for the treatment of sarcopenia, and can contribute to the diagnosis and prevention of sarcopenia. The negative association between FGF19 and sarcopenia was also confirmed by a recent cross-sectional study in Turkey of 88 older adults outpatient participants aged 65 years or older (9).

A number of literatures have shown that sarcopenia is related to inflammatory cytokines, but the existing studies are controversial on whether inflammatory factors play a positive or negative role in sarcopenia. Studies have shown that chronic inflammation leads to a loss of muscle mass by affecting protein synthesis and catabolism. However, most of the people in these studies were obese or had other chronic conditions, so the role of inflammatory factors in sarcopenia remains controversial (44). In a recent study, 299 Japanese residents (127 males and 172 females) participated in urban health check-ups. It was found that IL-6 is not an independent factor in sarcopenia (45). This conclusion is similar to ours. A recent community sarcopenia study in Taiwan also found no significant correlation between serum IL-6 levels and ASMI, handgrip strength, or gait speed (46). IL-6 is an effective regulator of human fat metabolism, which can increase lipolysis and fat oxidation. IL-6 acts in an anti-inflammatory way during muscle contraction. Because IL-6 has pro-inflammatory and anti-inflammatory effects, and the IL-6 secreted into the blood cannot be determined whether it is derived from muscle tissue (47).

The role of TNF-α in sarcopenia involves complex biological mechanisms. While elevated TNF-α classically promotes muscle catabolism via NF-κB-mediated proteolysis and IRS-1 suppression (48, 49), clinical studies (including our data) paradoxically report lower TNF-α levels in sarcopenic individuals, with multivariate analyses identifying hypo-TNF-α as an independent risk factor (45, 50). This apparent contradiction may stem from: (1) Malnutrition override: Severely depressed TNF-α could signal protein-energy wasting, exacerbating anabolic resistance; (2) Biphasic regulation: Extreme high/low TNF-α may both disrupt homeostasis, akin to its dual roles in tissue repair vs. degeneration; or (3) Inflammaging exhaustion: Late-stage sarcopenia may deplete inflammatory reserves. Critically, catabolic pathways (e.g., NF-κB/MuRF-1) remain activatable by alternative stimuli even when TNF-α is low. Further mechanistic studies must resolve these mechanisms.

There are some limitations to our study. First, as a single-center, cross-sectional study, We cannot distinguish whether changes in biomarkers cause sarcopenia, or sarcopenia leads to biomarker alterations, or whether other factors jointly contribute. Therefore, further longitudinal and multi-center collaborative studies are needed to validate our findings and explore the potential causal relationships between these biomarkers and sarcopenia. Secondly, because the participants came from rural communities, most of whom may have different occupations and living standards than older people in urban environments, our findings have limitations in generalizability and may not be directly applicable to Chinese urban older adults or rural settings in other regions.

Additionally, there are some unmeasured or unknown confounding factors that may affect the results of the study. Sarcopenia is an emerging and complex disease, and its influencing factors and pathogenesis are complex and diverse. Therefore, it is impossible to make a definitive diagnosis of sarcopenia through a single serum marker. Although key covariates (age, sex, BMI, percent body fat) were included in the multivariable regression models to account for their potential influence, several clinically relevant confounders remain unaccounted for. As highlighted in a recent review (51), lifestyle factors (physical inactivity, smoking, alcohol consumption, abnormal sleep duration patterns), socioeconomic factors (education, income, healthcare access), and malnutrition may concurrently influence biomarker levels and the risk of sarcopenia. However, these associations are primarily derived from non-prospective observational studies, and therefore the overall quality of evidence is low, as noted in the review. Therefore, further high-quality longitudinal cohort studies are warranted to elucidate the etiological basis of sarcopenia, thereby providing a foundation for improving strategies for its prevention, diagnosis, and intervention.

Another, the lack of investigation of the clinical characteristics of the participants with noncommunicable diseases is also a limitation of this study, and the noncommunicable diseases of the participants may also affect the conclusions of the study. In addition, this is a cross-sectional, single-center study that cannot definitively establish a causal relationship between serum biomarkers and sarcopenia. Although our exclusion criteria eliminated participants with specific disease states (malignant tumors, myocardial infarction or severe heart failure, renal failure during hemodialysis, severe mental illnesses such as bipolar disorder, schizophrenia, or mobility impairment), this study has not comprehensively analyzed the clinical characteristics of participants with specific non-communicable diseases (e.g., diabetes, cardiovascular disease, obesity, chronic obstructive pulmonary disease) or their potential influence on biomarker levels or sarcopenia associations. These conditions may act as confounders or effect modifiers affecting our results. Future studies should specifically explore sarcopenia-related biomarker profiles in comorbid states.

## Conclusion

This study shows that the prevalence of sarcopenia in rural older adults in China is 25.4%, 20.7% for men and 28.5% for women. BMI, serum ALB, FGF19, and TNF-α levels were lower in the older adults population with sarcopenia, while GDF11 was higher in the serum of patients with sarcopenia. These findings carry significant clinical implications: accessible indicators like BMI and ALB enable preliminary screening and risk stratification in rural communities, where low values signal higher sarcopenia risk and help identify nutritionally compromised individuals; concurrently, serum biomarkers such as elevated GDF11 and reduced FGF19 show promise for precision diagnostics and risk prediction, guiding future blood-based early-screening tools.

## Data Availability

The original contributions presented in the study are included in the article/Supplementary Material. Further inquiries can be directed to the corresponding authors.
